# The COVID-19 Vaccine: Trust, doubt, and hope for a future beyond the pandemic in Germany

**DOI:** 10.1371/journal.pone.0266659

**Published:** 2022-04-07

**Authors:** Amelia Fiske, Franziska Schönweitz, Johanna Eichinger, Bettina Zimmermann, Nora Hangel, Anna Sierawska, Stuart McLennan, Alena Buyx

**Affiliations:** 1 Institute of History and Ethics in Medicine, TUM School of Medicine, Technical University of Munich, Munich, Germany; 2 Institute for Biomedical Ethics, University of Basel, Basel, Switzerland; Public Library of Science, UNITED STATES

## Abstract

Public perceptions of COVID-19 vaccines are critical in reaching protective levels of herd immunity. Vaccine skepticism has always been relatively high in Germany, and surveys suggest that over the course of the pandemic, enthusiasm for the COVID-19 vaccine has dropped. Looking at the period just prior to the approval of the Pfizer/BioNTech and Moderna vaccines in Germany in the latter half of 2020, this paper aims to assess the reasons for and against COVID-19 vaccine uptake among residents of Germany, and to provide in-depth qualitative data to better understand and address concerns surrounding the safety and efficacy of a COVID-19 vaccine. Our findings indicate that there is widespread trust in German institutions and health experts to provide a safe vaccine for those who need it most. However, interviewees also point to the need for more information and the centrality of support from trusted medical authorities in making individual vaccination decisions. We also present the complexity of individual positions on vaccination, and suggest that vaccine hesitancy in relation to COVID-19 needs to be understood as a nuanced, and socially malleable, territory. This indicates that the goal of a vaccination campaign is not only achieving ‘herd immunity,’ but also a social endorsement of the collaborative effort that is required for a vaccine to be successful.

## Introduction

The COVID-19 pandemic has dramatically changed daily life for people across the globe. With more than 303 Million infections globally, and over 7.4 Million in Germany, the toll of the pandemic continues to rise [1]. Finally, at the end of 2020 it seemed that good news was on the horizon: a number of vaccines against the COVID-19 virus had been developed. In November 2020, Pfizer/ BioNTech reported promising results from the clinical trials of their vaccine, with 95% efficacy. These results were remarkably similar to the initial findings released by Moderna regarding their vaccine [2], with at least 58 other vaccine candidates in clinical trials on humans as of December 7, 2020 [3]. Both the UK and the US approved and began vaccine distribution in December of that year. However, to achieve protective population immunity (herd immunity) [4], more than 70% of the population needs to be vaccinated, raising critical questions around trust in governments, just distribution of the vaccine, and a continued societal commitment to preventative measures such as masks, hand hygiene, and physical distancing. It was clear early on that public views on the vaccine would prove critical in vaccine uptake. As the pandemic continues, it remains essential that the public is actively engaged in conversations around the risks and benefits of Covid-19 vaccines [5].

Existing surveys on public perceptions of the COVID-19 vaccine in 2020 suggested that those with an intention to get vaccinated hovered around 70%. A global survey of potential COVID-19 vaccine acceptance rates conducted in June 2020 found that 71.5% of participants across 19 countries reported being very or somewhat likely to get vaccinated, however with differences ranging from almost 90 to 55% [6]. A survey of European countries in April found that 73.9% of participants were willing to be vaccinated [7], however by June that number had dropped to 68% [8]. Similar surveys confirmed these findings, with 77.6% of respondents in France indicating their intention to get vaccinated [9]; 91.3% in China [10]; and 64% of respondents in the UK [11]. Surveys in the US ranged between 57% [12] to 69% [13]. Factors preventing vaccination included concerns about safety and efficacy of the vaccine, side-effects, a lack of trust, social norms surrounding vaccination, mis-information, and ability to access the vaccination [14]. Among European participants who were unsure about getting vaccinated, the most significant concern cited was potential side effects [7].

Drawing on data collected as part of a qualitative, longitudinal study seeking to understand the nature of solidaristic practices during the pandemic, this paper aims to assess the reasons for and against COVID-19 vaccine uptake in Germany and to provide in-depth qualitative data to understand and address concerns surrounding the safety and efficacy of a COVID-19 vaccine. Specifically, we are looking at the period in the pandemic just prior to the approval of the Pfizer/BioNTech and Moderna vaccines in Germany in the latter half of 2020. At that point, Germany had performed relatively well during the COVID-19 pandemic, reporting lower death rates and case numbers than other European countries [1]. Nonetheless, vaccine skepticism has always been relatively high in Germany [15, 16]. Of those who opposed vaccination in the European-wide survey from April 2020, the largest rates were in Germany and France [7]. A number of surveys found that in early summer 2020, only half of the German public would get the COVID-19 vaccine [17]. In another representative survey from November 2020, 42% of the population did not believe that their personal health was endangered by the virus [18]. In Germany, the drop in vaccination readiness was particularly pronounced compared to other European nations, decreasing from 70 to 61% between April and June. Further regional divisions were also apparent: 67% of people in the northern state of Lower Saxony said they would get vaccinated, while only 52% of those in the southern state of Bavaria said they would get vaccinated [8]. A survey reported in *Der Tagesspiegel* found that the speed of vaccine development led many to question whether vaccination was safe. Taken together, these finding suggest that there is a need for more-in depth understandings of public views of the COVID-19 vaccine and the concert of factors that affect willingness to be vaccinated.

## Methods

As part of a qualitative, longitudinal consortium study into “Solidarity in times of pandemics,” we conducted semi-structured interviews in Germany. The consortium, comprised of 9 European countries, was formed at the beginning of the COVID-19 pandemic in order to explore peoples’ experiences during the pandemic, with particular attention to practices relating to solidarity [19].

Participants were recruited through online advertisement on university websites, social media networks, and through snowball sampling in April 2020. All above 18 and living in Germany were eligible to participate. Participants were recruited with attention to a range of different demographics, including age, gender, income, household structure, geographic area, education, and employment (Table 1). The study was approved by the Technical University of Munich’s ethics committee (no. 208/20 S).

**Table 1:** Demographic distribution of participants in Germany.

Category	T1	T2
**Age **		
18–30	9 (20%)	7 (17%)
31–45	19 (41%)	18 (43%)
46–60	5 (11%)	4 (9%)
61–70	8 (17%)	8 (19%)
70+	5 (11%)	5 (12%)
**Gender **		
Female	25 (54%)	22 (52%)
Male	21 (46%)	20 (48%)
Other	0 (0%)	0 (0%)
**Household **		
Single	13 (28%)	13 (31%)
Couple	16 (35%)	15 (35%)
Living with child/children under 12	8 (17%)	6 (14%)
Living with child/children 12+	4 (9%)	4 (10%)
other	5 (11%)	4 (10%)
**Geographic Location**		
Big town (e.g. capital, +500k)	22 (48%)	22 (52%)
Medium/small town	12 (26%)	10 (24%)
Rural (e.g. village)	12 (26%)	10 (24%)
**Employment status **		
Employed (long-term contract)	24 (52%)	24 (57%)
Self-employed	4 (9%)	4 (9%)
Employed (short-term/precarious contract) Precarious	0 (0%)	0 (0%)
Unemployed	4 (9%)	2 (5%)
Retired	10 (21%)	10 (24%)
other	4 (9%)	2 (5%)
**Education level **		
Less than 10 years	2 (4%)	2 (5%)
10–14 years (e.g. high school diploma)	16 (35%)	13 (31%)
Higher education	28 (61%)	27 (64%)
**Household net income **		
Up to 1,400€/month	5 (11%)	2 (5%)
1,401–3,000€/month	14 (30%)	15 (36%)
More than 3,000€/month	27 (59%)	25 (59%)
**Total **	**46 **	** 42**

Interviewers explained the design and intent of the SolPan project to all participants, who then gave consent to participate orally. Interviews ranged from 30 to 45 minutes in length, and were conducted in German with the exception of one interview which was held in English. Interviews were recorded on a digital recorder or using a GDPR-compliant video chat recorder. Only audio material was stored. The interviews were transcribed and subsequently pseudonymized; due to the size of the SolPan project, interview transcripts were not returned to participants for comment.

The interviews were conducted in two periods (T1 and T2) approximately 6 months apart, the first covering the “lock down” period in Germany in April 2020 (6 April-6 May 2020), and the second in October 2020 (2–28 October). 46 individuals participated in T1, and 42 participated in T2. Four individuals elected not to participate in T2, or were not reachable for interviews at the time. Demographic information was collected at the conclusion of the first interview (T1), and interviewing for T1 concluded once the authors collectively agreed that different demographic categories were reasonably represented. Most interviewers interviewed the same individuals in T1 as in T2. Some interviewers took notes during the interviews, which were subsequently destroyed in accordance with data protection. The interviewers had no prior relationship with participants.

The interview guides used in this study for T1 and T2 were generated by a group of researchers in the SolPan consortium, and iteratively edited across the nine country teams in order to generate a final guide [20, 21]. Country teams were encouraged to follow the guide, but free to add in some country-specific questions. The interview guides were pilot tested prior to implementation. All individuals in T2 were asked a series of questions about vaccines (Table 2). Although there was no specific question regarding vaccines during the T1 interviews, nearly half of the respondents [21] spontaneously brought it up. The T1 data on vaccines is reported in the “Differences in hopes for the vaccine over the course of the pandemic” section; the rest of the results are drawn from T2 data.

**Table 2:** Interview questions asked in T2 about vaccines.

Questions asked:
There have been many reports about the development of vaccines against COVID-19. What is your opinion on this?
If there was a vaccine against COVID-19, under which conditions would you get vaccinated and under which not?
How would you proceed to get the vaccination?
If vaccines are scarce, who do you think should receive them first?
Who do you trust most when it comes to vaccine information?
Do you have a different attitude towards the COVID-19 vaccination than to other vaccines, e.g. seasonal flu vaccination?

All authors conducted the interviews as part of the SolPan project, and most of the authors completed interviews in Germany [AF; FS; BZ; NH; AS; AB]. Interviewers were trained in qualitative interview techniques; the majority of the authors have advanced degrees in social science disciplines and specialize in qualitative research and design. The majority of the interviewers identify as female, and all were employed by the Institute for History and Ethics in Medicine at the Technical University of Munich at the time interviews were conducted.

The interviews were coded using an inductively generated coding scheme created using a grounded theory approach [22] as part of the SolPan consortium. Eight members of the SolPan research team coded the T1 and T2 data using Atlas.ti software (including five of the authors); all authors have access to this dataset [21]. Coding was checked by a second researcher for consistency. Relevant text passages were extracted via the Atlas.ti query function and analyzed inductively. Responses were also analyzed with respect to demographic information in order to ascertain if different positions in relation to vaccination tended to be associated with age, gender, household composition, geographic location, employment status, education level, and income level. No notable differences emerged in this regard, and as such we do not address demographic characteristics in the results section.

## Results

### Willingness to get vaccinated against COVID-19

Many respondents expressed no hesitation in getting vaccinated in the second phase of interviews (T2): “Oh, when there is one, then I would get vaccinated immediately, no question,” (NH13). Others qualified their answers by stating that they would get vaccinated once a vaccine was proven to be effective, and had gone through necessary testing and safety procedures. Some suggested that the COVID-19 vaccine could become something that was considered routine, such as like vaccinating children against measles, indicating that they would not put any conditions on the vaccine but rather, that they simply trusted “our system,” and would “get vaccinated without hesitation,” (BZ04).

Some respondents indicated that they would prefer to wait until after a COVID-19 vaccine was released and had been administered to other people in order to evaluate if it was safe enough to receive. This included respondents who were generally positive about vaccination and COVID-19 vaccines in particular, as well as those who were more skeptical about vaccination. For example, one individual noted that they would not want to be the first in line in this case, but “I would just wait for a while and see how others are doing after the vaccination” (FS01). For some this meant waiting a few months, while for others this meant waiting years, or even up to a decade–before they would feel there had been sufficient evidence to say that the vaccine was safe.

The issue of uncertainty surrounding the efficacy and safety of the vaccine weighed heavily among some respondents. One individual noted that this involved a trade-off when considering personal safety: “if the vaccine hasn’t been tested so incredibly well or hasn’t been tested for so long, I sometimes ask myself, do I want to be the first to do that?” (NH11). Others cited the length of time that vaccines normally take to be developed, and contrasted this to the relatively fast development of the COVID-19 vaccine, questioning whether it would be really as safe as other vaccines. In a telling example, one individual compared the COVID-19 vaccine to the way she would evaluate any kind of new medical treatment. Citing the relative newness of laser eye surgery two decades ago, she said she now felt confident that it was safe because so many had already undergone it. Despite not having any specific concerns with the COVID-19 vaccine, “I feel like I would like to wait for half a year more, even if it meant a lockdown for me here or something, just to be really sure that the risk of being vaccinated is so low that it’s safe to do it,” (AF01).

Some individuals reported that they were undecided, or that they would choose not to get vaccinated against COVID-19. In most cases, respondents cited concerns surrounding the safety and speed at which the vaccine had been developed. Some noted that they would like to research the topic before they could decide (BZ07).

I would not get vaccinated […] Because I am highly skeptical that this is safe […] and it’s so hastily cobbled together that I wouldn’t really trust it to indeed be efficacious. Then you always have to see what the side effects are. I could imagine that the side effects won’t only be minor. Yes. But I’m not a great fan of vaccination anyway. I have been vaccinated when it is unavoidable, but I do not jump on every vaccination bandwagon. (BZ03)

Others were vehement that they felt that the standards of vaccine development had been eclipsed. This reflected a broader pattern among respondents: opposition to the COVID-19 vaccine did not fall on simple distinctions of pro- vs anti-vaccine groups. However, the sentiment that normal procedures had been circumvented in the rush for a vaccine was common.

For some individuals, prior personal experience with a bad side effect from a flu vaccine shaped their evaluation. Several wanted to discuss vaccination with their physician. For many, the fear of side effects was generalized and not about a specific health risk, yet many cited a desire to “weigh” the risks and benefits in their individual case. The decision to vaccinate was closely linked to the personal perception of risk posed by COVID-19. Several individuals concluded that the potential risks of a vaccine were not outweighed by those of the pandemic, given that they were personally in good health.

The difference is the probability that if I fell ill with Covid-19, would I have permanent damage or die from it? This is what is being preached every day. In my view, this is not necessarily the case. The risk or even the effort to be vaccinated against it is, in my view, not given at all. And why then with a substance, which was never tested, whose effectiveness is not at all proven? I mean, in the best case it simply does not work … [So] is it worth the risk? No, not at all. Above all, I am at an age where I have nothing to fear. I do not have asthma, nor do I have any other diseases that are proven to be problematic in connection with an infection […] As I said, I am not afraid of getting infected [with COVID-19]. (BZ05)

### Motivations for getting vaccinated against COVID-19

For those individuals who were in favor of vaccination in the October interviews, motivations centered broadly around a wish for the pandemic to end, in which both personal and collective benefits were intertwined. As one respondent put it, the reason was simple: “not to get sick and of course not to make others sick,” (NH10). This individual went on to explain that they were afraid of catching COVID-19, and had seen how contagious the disease was and the effects of it across society.

For other individuals, vaccination offered a welcome reprieve from the restrictions imposed by the pandemic, potentially allowing freedom of movement and a degree of normality again. Another respondent noted that their principle motivation for vaccination would be self-protection, and while they did not like getting vaccinated, they would be willing to if it meant that social life would return to normal again. Others reiterated the possibility of certain restrictions being relaxed, but also noted that the individual choice to get vaccinated could serve as an example for others to follow.

For some people, the vaccine was the ultimate answer to the pandemic. As one individual noted, once everyone has been vaccinated, “the problem is over.” (NH07). Yet the sense of optimism was tempered in most responses, with others less hopeful that the risk of COVID-19 could ever be eliminated, noting that vaccination was nonetheless important in order to minimize that risk. Until a vaccine was available, another respondent noted, their behavior (mask wearing, reducing contacts) would not change.

### Information on the COVID-19 vaccination and trusted sources

There are three principle institutions charged with ensuring the safe delivery of the future COVID-19 vaccine in Germany: the Robert Koch Institute (RKI) which is responsible for disease control and prevention; the Paul Ehrlich Institute (PEI) which is responsible for testing the safety of vaccines; and the Standing Commission on Vaccination (STIKO) which provides official recommendations on vaccination schedules. The German Ethics Council (DER) and the National Academy of Sciences Leopoldina have also been involved with STIKO in proposing criteria for a fair prioritization of COVID-19 vaccines.

Across the October interviews, one of the most common sentiments was broad faith in German institutions such as the RKI, the PEI and STIKO to get the COVID-19 vaccine right. It was unthinkable to most that German institutions would approve a questionable vaccine. Respondents felt that these institutions would not be rushed into issuing a vaccine. This sentiment was repeated many times, with another interviewee indicating that if the vaccine was ‘mature’ that it could be given without hesitation. When asked how they would know, the respondent replied that they would wait for an official recommendation from the German federal government or the RKI. The responses indicated significant support and trust in the institutions that were in place to ensure safety.

If a vaccine is available and is recommended, then I would do so. Yes, as I said, this would not be possible in Germany anyway, because there is a drug law and a medical device regulation for this […] There are simply corresponding regulations and laws that a vaccine has to follow and if the STIKO recommendation […] advises to do so, then I would take it. It cannot be otherwise. There is no way it can be reasonably brought to market without having been adequately tested. (FS04)

Central to these conceptions of trust was straightforward and transparent communication with the public. For other respondents, faith in the process extended beyond Germany to the EU.

The widespread confidence in German institutions was sharply contrasted with mistrust for the vaccine development in other countries. Respondents indicated that their support for a COVID-19 vaccine was contingent on the domestic approval, without the perception of foreign influence. Another individual described their understanding that the vaccine trials in Russia had not been properly vetted. They contrasted this to the process in Germany. In this sense, their support for the COVID-19 vaccine rested entirely on the approval of German authorities.

In addition to widespread trust in experts and expert institutions in Germany, for many respondents it was important to discuss their decision with trusted contacts, such as their physician, or others whom they felt were knowledgeable on the subject. References to discussing the RKI recommendations with family doctors were common. These responses suggested that the interpersonal network of individuals is central to vaccination decisions, and in particular the centrality of the general practitioner, or *Hausarzt*.

Some individuals expressed a desire for more information about the vaccine. This included general questions about safety and side effects, as well as specific questions relating to the mechanism by which it would create immunity or how long acquired immunity would last. Others noted that they would like to be able to personally evaluate the clinical trial data.

### Comparison to the annual influenza vaccine

In order to assess if there were differences in sentiment relating to the COVID-19 vaccine and the annual flu vaccine, respondents were asked in the October interviews if they considered the two to be different. For some respondents, there was absolutely no difference, noting that they regularly got their flu shot. Some said that they regularly get a flu vaccine, and likewise the COVID-19 vaccine, as a means of contributing to basic herd immunity. This was echoed by those who noted that the rationale was the same for both vaccines. For some, the reverse was true: they never got flu vaccines and had no intention of getting the COVID-19 vaccine either: “I have the same attitude to the Covid-19 vaccination as I do to the flu vaccination. So, I am not vaccinated against influenza because I do not belong to the risk group, in my opinion. Why should I get vaccinated now?” (NH18).

Other respondents felt that the COVID-19 vaccine was decidedly more urgent than the flu vaccine, citing the increased risk posed by COVID-19. Some noted that COVID-19 was a greater threat on both a personal and societal level, with one respondent noting that vaccination was important because they would also be protecting others in the process. The fact that one disease was a pandemic with far greater consequences than the flu was a critical factor for many. Notably this included an individual who had previously had a bad experience with a flu vaccination.

For a number of individuals, the ongoing COVID-19 pandemic prompted them to get a flu vaccination for the first time. Some cited the dual risk posed to the healthcare system for the annual flu wave and COVID-19 pandemic, including one individual who described a physician colleague explaining the risk of overload, and another who noted, “Okay, then at least you are halfway secure in that case and you would not overload the [health care] system if you get infected with a more serious disease,” (NH06). Others who had dismissed the annual flu vaccine for years changed their minds after seeing the effects of the pandemic.

### Prioritization of a potential COVID-19 vaccine

Individuals were asked how they felt a future COVID-19 vaccine should be distributed, assuming that the vaccine would initially be rationed. Common sentiments included the need to prioritize those who were at greatest risk of dying from a COVID-19 infection, whether due to pre-existing health conditions or age. Many individuals felt that those who were “system-relevant” workers, and medical professionals (in particular doctors, nurses, and nursing home staff) should be prioritized in order to protect the health care system and necessary public services. Other groups that were mentioned with less frequency included teachers, or individuals who worked in settings where there was no possibility of physical distancing. Others simply deferred to expert or scientific guidance on the matter, suggesting that they trusted that the vaccine would be appropriately distributed.

Interviewees answered the questions regarding prioritization largely independently from their personal views of the COVID-19 vaccine, meaning that even those who stated that they would not get a COVID-19 vaccine still described a need to prioritize those most at need.

Interestingly, none of the respondents self-identified as being in a risk group for the vaccine, despite the fact that several were over 70 years of age. Some, including those who were over 60 or had pre-existing conditions, noted that they did not expect to be prioritized and that they were fine with that. This included explicit expressions of solidarity with others who needed the vaccine more urgently, even if the recipient was younger and theoretically less at-risk health wise:

I’m now [age 60+], I would understand if someone told me that, ‘listen, it will be your turn only in the third or fourth round.’ Honestly. Because I see that there are people who need it much more urgently. And I see what happens when the daycare is closed, when my daughter can’t go to work. … I am older but not yet one of the very old–it is not something I see as a priority. (NH10)

A similar sentiment was expressed by another individual who was also over 60 years of age, who said that despite their desire to be vaccinated, they fully understood the need to distribute the vaccine to those who were most at risk.

There was widespread sentiment that those who were most at risk should be the first to get the vaccine. However, for those who were concerned about potential safety and unknown side effects of the vaccine, the notion that those most at risk would be exposed first–when the vaccine was perceived to be the ‘least’ safe–was described as counterintuitive. One individual wondered, “Sure, I would like to say ‘Of course the ones who need it most, the elderly.’ But what if they then have much worse side effects with such a vaccination?” (NH06). Another questioned the idea of giving the vaccine first to the elderly, under the same rationale. Finally, one respondent said that they would prefer to wait to evaluate how the vaccine was received by the general public, described the difficulty of prioritization questions as a result of this seeming contradiction.

### Differences in hopes for the vaccine over the course of the pandemic

While the first round of interviews conducted in April did not explicitly ask about a vaccine for COVID-19, approximately half [21] of the respondents spontaneously brought up the topic. The majority of these comments were on the issue of whether or not a vaccine would ‘resolve’ the pandemic, with 14 people speaking of the vaccine as the only ‘cure’ for the pandemic. Among these respondents, there was a shared hope that within the year a vaccine would be developed and life could return to normal. In addition, during the first round of interviews, some questioned whether or not the vaccine development process would be transparent enough. Only three respondents noted during the T1 interviews that a vaccine would not be a cure, speculating that the virus would continue to mutate or that the vaccine would not be tolerated by everyone.

Six months later, the sentiments of respondents had shifted such that few people were discussing the vaccine as a straightforward cure for the pandemic. Instead, expectations were tempered and some respondents were resigned that a vaccine was not the only solution to the problems brought by the pandemic. Some respondents noted that as a society, we will have to deal with the COVID-19 as with other (recurring) viruses like the flu. In a similar sentiment, one respondent described the future of ‘waves’ of COVID outbreaks, such that eventually “we will learn to live with this disease at some point,” (BZ03).

Many did not think that there would be a dramatic change with the arrival of the vaccine: “I don’t know whether it will be the panacea after all […] I don’t think the situation will relax over the winter,” (NH15). By the second phase, when vaccine development was a much closer reality, respondents were far more concerned with issues of safety, efficacy, and logistics than they were when the vaccine was not yet available. As one person put it, “Last time I don’t remember how optimistic I was then. But now today I would say that I don’t think that [the vaccine] will change much.” (NH08).

## Discussion

While many participants had concerns regarding the safety of the vaccine due to the rapid timeframe in which it was developed, these doubts were significantly buffered by widespread trust in German health authorities. This maps well against existing findings that suggest that trust in authorities is comparatively high in Germany [23]. However, attitudes towards the COVID-19 vaccine were clearly shifting with the nearly 10% drop in willingness to be vaccinated between April and June 2020. Further, significant regional differences in willingness to be vaccinated suggest that some parts of Germany had levels of public acceptance as low as 50% [8], possibly resulting in uneven vaccination progress across the country. Reading the national survey results together with the concerns raised in our qualitative interviews indicates that it is important that the apparent trust in health authorities not be taken for granted. Trust in authorities does not necessarily translate to voluntarily getting vaccinated in a timely fashion. Reflecting back on these findings prior to the availability of vaccines, clear, transparent discussions between experts and the public emerge as critical in answering outstanding questions about the vaccine, potential side-effects, and in particular, the process that went into developing, testing, and certifying the vaccine. This remains true today, as questions arise with the need for booster shots, mix-and-match boosters, and encouraging vaccine uptake among the unvaccinated. This is especially true in light of the confusion regarding the safety and recommended age requirements in the case of the AstraZeneca and Jansen vaccines in Germany [24, 25].

The responses from the interviews illustrate that a vaccine never operates in a vacuum. No vaccine is 100% effective, and there will always be some members of a population that cannot be vaccinated because of medical reasons [26]. As Thomas Mertens of the STIKO indicated, both infrastructure and a high public acceptance of vaccination are key for rolling out a new vaccine and reaching a high enough level of immunity to significantly change the course of the pandemic [27]. Thus, any conception of ‘success’ in relation to vaccination needs to include strong public health infrastructure, widespread public trust in the structural conditions guiding vaccine development, and clear communication between health authorities and the public surrounding the necessity and function of the vaccine, the plans for distribution, and any potential side effects. Our findings point to the crucial role that general practice doctors and other local professionals that individuals may turn to for vaccine advice may play in this vaccination infrastructure. This is particularly important given a recent study by the COSMO project that found that vaccination readiness was lowest in the health worker group, with levels lower than the general population, those with chronic diseases and the elderly [28]. By contrast, a separate survey found that medical professional willingness to be vaccinated against COVID-19 was quite high, at 91%, and was higher than medical professional readiness for the annual flu vaccine [29]. Despite these contradictory survey results, our results show that health professionals are trusted sources of information in their communities and their perceptions may affect public readiness towards vaccination. It is clear the embedding of individuals within networks of support that allow them to evaluate expert information is critical. One of the most resounding findings that emerges from this study is that vaccination is a societal effort; this suggests that the goal is not only achieving ‘herd immunity,’ but also a social endorsement of the collaborative effort that is required for a vaccine to be successful.

Among those who expressed significant doubts or skepticism about the vaccine, the reasons given usually related to the speed of the development process and the uncertainty of the evidence. However, it is important to note that the interviews were conducted before the initial release of the clinical trial data at 95% efficacy [2]. In some cases, individual responses suggested misconceptions, indicating that a clear communication campaign on the function and testing of the vaccine is always necessary. In many cases, the concerns were more general than specific. Some respondents seemed to be unsettled by the news of vaccines testing in Russia or the US, with the suggestion that due process was not being followed. Building public confidence in COVID-19 vaccination, and in particular the German and European process that led up to the vaccination campaign, is central to addressing fears around vaccination. This seems to have been additionally important given the novel mechanism of the major vaccines.

Individual positions on vaccination are wide-ranging. Significant variation within the category of ‘vaccine hesitancy’ may be glossed over in national surveys [30]. It is clear in our results that it is not productive to think of distinct ‘camps’ of those who are strictly for or against the COVID-19 vaccine. Further, there are significant differences among those who may be labelled as “vaccine-hesitant,’ and it is important to understand this hesitancy as complex and context specific, and subject to change based on time, place, and the vaccine in question [31]. Our results suggest that for many individuals it is important to feel that they have the information needed in order to evaluate the vaccine for themselves. Some individuals who had doubts about the safety of the COVID-19 vaccine went on to clarify that they were not “anti-vax.” Likewise, some who endorsed the vaccine went on to suggest that were not blindly following the vaccine “bandwagon.” In comparisons to the annual flu vaccine, many noted they had elected to get the annual flu vaccine due to the pandemic despite normally not getting it. This suggests that vaccine hesitancy in relation to the COVID-19 vaccine is a nuanced territory and will continue to shift as more evidence regarding the safety and efficacy of the vaccine emerges, in particular as the vaccine is distributed. However, it is important that COVID-19 vaccine hesitancy not be simply dismissed as across-the-board rejection of vaccination, and should be met with nuanced information combined with multiple levels of public and expert support. There will be no single solution to vaccine hesitancy in relation to COVID-19 [26].

Responses to the question on prioritization quickly reached a ‘saturation’ point, suggesting a broad sense of consensus surrounding vaccination prioritization based on individual risk and profession (e.g. for health professionals). These responses are largely consistent with the prioritization recommendations of the STIKO, which designated the first group to be vaccinated as those who have an increased risk of poor health outcomes due to underlying medical conditions, and the second group to as those who work in medical or long-term care facilities [32]. The significant overlap between interview responses and the Joint Position Paper guidelines point to widespread acceptance of this tiered proposal for distribution. Similarly, a national survey found that 93% of those surveyed agreed with the prioritization scheme for the vaccine [18]. Particularly interesting is that no one, including those who were over 70 years of age, self-identified as being in a risk group. This may mean that there was a disconnect between individual’s perceptions of their own personal risk in relation to COVID-19 and the prioritization guidelines’ understanding of risk. It is important moving forward that clear communication continues surrounding the specific risks posed by age, employment, or other forms of vulnerability in relation to COVID-19 and the need for vaccination.

The overlap between the Joint Commission’s position paper and our results shows that there was a shared understanding of stratified ‘social need’ in relation to the effects of the pandemic, and a willingness to wait so that those in need can have the vaccine first. However, we were unable to assess if people’s willingness to be vaccinated was tempered by the assumption that they would *not* be first in line, and therefore would be receiving the vaccine after it had already been administered to many others. One striking contrast was the doubts raised by some respondents regarding whether risk groups should receive the vaccine first, given the perception that the vaccine would be the ‘riskiest’ earlier in its distribution. Further, even those who doubted the safety of the vaccine still suggested similar priorities for distribution. No respondent–including those who said they would not get vaccinated–said that the vaccine should not be given at all. This suggests that there may be an important difference between individual risk assessment and societal risk assessment in relation to the COVID-19 vaccine. This is in line with results from a recent study on voluntary and mandatory vaccination, where four attitudes regarding the vaccination were identified, including those who would not voluntarily get vaccinated, but were in favor of mandatory vaccination [33]. That is to say, some may try to benefit from the decisions made by others and the same time would refrain from contributing themselves.

On the whole, hope in the vaccine was measured. In comparing responses across the six-month period between the T1 and T2 interviews, as a vaccine became more of a reality, more questions regarding efficacy, safety, and how long it would take to effect widespread change emerged. This suggests that there was a shared understanding that the vaccination campaign would take a significant amount of time and would not simply ‘resolve’ the pandemic overnight. It also means that public perceptions of the vaccine are malleable; concerns can be clearly addressed through transparent communication, and likewise, doubt about the safety and effectiveness of the vaccine could grow if left unanswered.

## Limitations

This was a qualitative study conducted with a relatively limited number of individuals in Germany, and thus the responses and conclusions are not representative. No differences in relation to demographic characteristics were discernible in the data analysis; it is possible that this is in part due to the limited number of participants in the study. Interviews were conducted in October 2020 before first results from COVID-19 vaccine trials were released, so the views presented are prior to the release of clinical trial data.

## Conclusions

Much of the existing public skepticism in Germany is related to the accelerated timeframe in which the COVID-19 vaccines was developed [34]. The findings from our research illustrate that many people did not have many specific concerns in relation to the vaccine’s safety, efficacy, and delivery, but rather held more generalized concerns about safety in relation to speed of development. Key steps were taken to assuage public concerns on this front, including the establishment of a monitoring and surveillance system at the RKI to follow the administration of the vaccine. In addition, we would advocate for continued, clear communication to explain the process of vaccination approval in Europe across national and local levels. More generalized concerns can be tackled with clear, transparent information that is readily accessible to the public, available in multiple languages, and tailored to specific demographic groups such as health professionals, high-risk patients, and young people. Specifically, our results suggest that a public explanation of how the standard safety procedures were enacted within the accelerated timeline of vaccine development is necessary in order to assuage fears of expediency being favored over safety.

Particular conditions emerged in the interviews that are important for health authorities to pay attention to, including the need for the independence of the German and European vaccine approval process. A second condition is that while trust is high in national institutions, people turn to local experts such as general practice physicians in order to assess if the vaccine is right for them. This means that vaccination efforts also depend significantly on this kind of ‘second-tier’ or informal support. These conversations, while out of view of the official vaccination program, may be particularly fruitful in answering questions and encouraging people to follow-through with an initial vaccination series or booster shots in the German context, as physicians may have an amplified effect on public readiness. As such it may be productive to have a national campaign that reaches out to health professionals and local leaders to provide them with clear information about the COVID-19 vaccine and strategies for discussing vaccine concerns with their patients or constituents. Finally, public health messaging should emphasize the relationship between individual vaccinations and the broader public good. This could be particularly helpful to support the overall solidaristic readiness we found to give those at greater risk priority.

Our findings provide a more in-depth understanding of how some individuals are both hesitant about the specifics of the vaccine but also supportive of those most in need receiving it first. This suggests the need for a nuanced approach to vaccine communication, and in particular, a differentiated response to vaccine hesitancy that addresses not only concerns surrounding safety and efficacy, but also the particular necessity of the vaccine in relation to the threat of the COVID-19 pandemic. Given that many respondents’ willingness to be vaccinated was explained in relation to their own perception of the threat that COVID-19 posed for their health, it continues to be necessary for health officials to emphasize that vaccination is not only about an individual benefit. Efforts need to be made to expand the individual risk calculus of catching COVID-19 versus vaccination to include the many societal benefits afforded by vaccination. Ultimately, we find that further reassurance from both national and local health professionals that any vaccine made available in Germany is safe and effective will continue to be needed, lest the public trust in COVID-19 vaccination wane to such an extent that vaccination efforts could be significantly compromised [7].

## Supporting information

S1 Appendix COREQ (COnsolidated criteria for REporting Qualitative research) checklist. (PDF)
